# The impact of cognitive distraction on gustatory perception in volunteers with obesity

**DOI:** 10.1038/s41598-024-64722-0

**Published:** 2024-06-20

**Authors:** Iryna Ruda, Deepak Charles Chellapandian, Jessica Freiherr

**Affiliations:** 1 Department of Psychiatry and Psychotherapy, Friedrich-Alexander-Universität Erlangen-Nürnberg, Schwabachanlage 6, 91054 Erlangen, Germany; 2 Sensory Analytics and Technologies, Fraunhofer Institute for Process Engineering and Packaging IVV, Giggenhauser Strasse 35, 85354 Freising, Germany

**Keywords:** Obesity, Gustatory perception, Distracted eating, Intensity, Pleasantness, Cognitive neuroscience, Gustatory system, Feeding behaviour, Obesity, Sensory processing

## Abstract

Obesity, a global health challenge, is influenced by biological, behavioral, socioeconomical, and environmental factors. In our technology-driven world, distracted eating is prevalent, yet neurocognitive mechanisms behind it remain poorly understood. This study targets individuals with overweight and obesity, exploring taste perception under distraction comprehensively. Participants formed two distinct groups based on their Body Mass Index (BMI), lean and overweight/obese. During the experiment participants received gustatory stimuli while playing a Tetris game of various difficulty levels. Participants rated taste intensity and pleasantness, with linear mixed models analyzing distraction effects. Results confirmed that high distraction levels reduced perception of taste intensity (*p* = 0.017) and taste pleasantness (*p* = 0.022), with variations influenced by gender and weight status. Individuals in the overweight/obese group exhibited most profound intensity changes during distraction (*p* = 0.01). Taste sensitivity ratings positively correlated with BMI interacting with gender (male r = 0.227, *p* < 0.001; female r = 0.101, *p* < 0.001). Overall across both groups, female participants demonstrated higher taste sensitivity compared to male participants (*p* < 0.001). This study highlights the impact of cognitive distraction during consumption on taste perception, particularly in relation to weight status and gender, underscoring their significant roles in this interplay.

## Introduction

Obesity presents a pressing global health challenge, affecting a continually growing number of individuals^1^. While the causes of obesity are intricate and multifaceted, one relatively underexplored factor contributing to its prevalence is the environment and context in which we consume food. In today’s fast-paced society, the increasing demand for multitasking often leads to food consumption occurring concurrently with attention-demanding cognitive activities. Food perception, a cornerstone of which is the concept of flavor, encompasses a complex interplay of multiple sensory modalities^2^. Among these modalities, the sense of taste plays a pivotal role in selecting nutritious and safe food sources, shaping food preferences, and influencing long-term eating behaviors^3,4^. Therefore, comprehending the impact of cognitive distraction on taste perception is of paramount importance.

Numerous studies have successfully demonstrated^5,6^ that distraction can result in increased food consumption, a behavior alarmingly associated with overconsumption and obesity, even observed in preschool children^7,8^. However, there remain certain limitations and controversies in the existing literature. It remains unclear which factors contributing to flavor and essential for food perception are affected by cognitive distraction. While some attempts have been made to examine the taste and smell systems—the primary determinants of food acceptance—under distraction, these efforts have generated varied and sometimes conflicting findings.

Certain studies have revealed a pattern where intensity ratings of various taste items tend to decrease when investigated under various types of cognitive load, in some cases, resulting in a preference for higher concentrations of taste solutions^9–11^. Notably, this effect of cognitive load is modulated by intensity of the stimuli, with more pronounced effects on taste ratings of strong rather than weak concentrations^11^. A corroborating study conducted by Liang et al.^10^ reported an effect of memory load on detection ratio for sweet and bitter solutions, with a decline in detection ratio being dependent on stimulus concentration and cognitive load effect is most prominent at concentrations around detection threshold. In contrast, Duif et al.^9^ observed a reduced perception of intensity in response to low-sweetness chocolate milk but not in the case of a high-sweetness drink, both at the behavioral and neuronal levels. Similarly, within the realm of olfaction, Hoffmann-Hensel et al.^12^ observed a decrease in the intensity of odors associated with low-caloric foods, while no discernible effect was noted for odors linked to high-caloric foods. Noteworthy, despite the wealth of studies, the understanding of how cognitive load impacts chemosensory perception remains fragmented and ambiguous. Adding to this body of knowledge, a recent study by Schadll et al.^13^ further substantiated the notion of changes in odor perception under conditions of distraction. Notably, this study advances the field by employing a more ecologically valid approach, utilizing a Tetris game as a means of cognitive load. Unlike memory tasks, Tetris engages visuospatial working memory, decision-making, strategic planning, motor dexterity, and other cognitive processes, offering a more comprehensive and realistic experience that mirrors the dynamic cognitive distractions encountered in everyday life situations.

It is crucial to note that many prior studies have employed complex multisensory food stimuli, which does not permit investigating effects of distraction on individual sensory processes like taste perception. Moreover, it has been suggested that effects observed in more complex food stimuli often including an active aroma component, may be explained by investigated distraction-induced changes in olfaction. Thus, a fundamental question arises: Is the gustatory system inherently susceptible to distraction, and if so, what are distraction-induced alterations of taste perception? To address this question, our study is designed to investigate the gustatory system in isolation from other sensory modalities, shedding light on its distinct performance under conditions of distraction.

Another critical gap in the field is the predominant focus on studies involving normal-weight adults. Despite the solid body of literature investigating alterations in individuals’ eating behavior and food reward responses in the context of obesity^14^, the intricate interplay between taste perception and body mass index (BMI) remains elusive. A recent comprehensive review underscores the presence of ample evidence indicating an association between obesity and taste alterations in adults^1^. However, it also emphasizes the inconsistent nature of the findings within the literature. For example, when comparing threshold of taste perception among individuals with obesity to those with normal weight, divergent trends emerge; including reports that participants with obesity show heightened taste sensitivity^15^ and a preference for stronger taste concentrations^16^, display diminished taste perception for some flavors or no differences between the groups reported^1^.

These alterations of taste perception at the behavioral levels, are potentially orchestrated by neural changes in reward and motivation systems, as well as in executive control and self-regulation systems, that were observed in subjects with obesity with regards to food^17^.

These findings raise a compelling question: will the known features of taste perception in obesity translate into distinct influences of distraction on the gustatory system in individuals affected by overweight and obesity? To answer this question, we systematically compared changes in perception of taste intensity between participants of different weight status, utilizing an ecologically valid Tetris game as a distraction paradigm^13^. Going beyond perceived intensity, we aimed to investigate how distraction affects the pleasantness of taste perception. Despite the critical role of pleasantness in influencing food perception and moderating food intake, it has received limited attention in prior research. Consequently, it remains unclear whether pleasantness is influenced by distraction and whether this influence varies based on individual characteristics. While our study does not have a predetermined hypothesis regarding the direction of pleasantness changes due to the scarcity of prior research, we anticipate that it is indeed influenced by distraction. Advancing our understanding of the factors shaping taste perception, including pleasantness, contributes to a deeper comprehension of the neurocognitive mechanisms underpinning distracted eating.

## Materials and methods

This manuscript used the CONSORT reporting guidelines^18^.

### Participants

Fifty-eight self-reported non-smokers participated in the study. Prior to the testing, participants filled out an online questionnaire used to screen for several exclusion criteria. If a participant scored more than 19 on the Beck Depression Inventory (BDI)^19^, participation in the study was not granted. Furthermore, given a known link between taste and smell perception and to control for potentially influential differences between the groups, 16-items Sniffin´ Sticks identification (SSI) test^20^ was used to confirm normosmia of all participants (Table 1). If a participant scored less than 11 out of 16, their participation in the study was terminated.

**Table 1:** Descriptive statistics of the study sample and group comparison.

	Lean	Overweight/Obese	*p* value
N	30	28	
Gender	15 males	16 males	
Age	26.6	28.8	0.28
BMI	22.1	30.0	**< 0.001**
Sniffin´ sticks identification test	13.1	13.0	0.65
BDI	6.1	4.3	0.57

Bold font represents statistically significant differences between both groups.

On the day of the experiment, participants` Body Mass Index (BMI) was calculated and, if required, self-reported values were corrected. Height was measured without shoes on and to access the participants weight a 100 g precision floor scale was used. We aimed to have two sufficiently different groups based on participants` BMI. The inclusion BMI range was set to be more than 18.5 kg/m^2^ but less than 24.9 kg/m^2^ for the lean group and above 25 kg/m^2^ but below 40 kg/m^2^ for the overweight/obese group.

Participants were not invited to the experiment if they had food allergies to the substances used in the study, were pregnant or breastfeeding or taking medication impacting their taste perception. Furthermore, participants with self-reported taste/smell disorders, depression, diabetes, oral/nasal/sinus infections, hypothyroidism (not treated) and smokers were not granted participation in the study. We also set a BMI cut-off of 40 kg/m^2^ due to difficulty in recruiting participants with higher BMIs who did not have other medical conditions (e.g. metabolic syndrom, bariatric surgery) that could impact the study results. Additionally, the armrest-chair used had weight and size restrictions for comfortable seating.

In total, 8 participants were excluded from the study: 2 failed the SSI test, 3 scored above the inclusion threshold of BDI, 1 had an allergy related to the study stimuli, and 2 had a BMI above 40 kg/m^2^. A normal-weight group consisted of 30 (15 males) subjects (mean age = 26.7 years, standard deviation (SD) = 6.4, range = 19–43 years; mean BMI 22.2 kg/m^2^, SD = 1.7 and range 19.5–24.8 kg/m^2^); another 28 (16 males) subjects formed an overweight/obese group (mean age = 29 years, SD = 6.7 and range 19–48 years; mean BMI = 30.0 kg/m^2^, SD = 1.7 and range 25–39 kg/m^2^)(see Table 1) . Among the participants in the overweight/obese group there were 5 female (mean BMI = 34.8, SD = 3.8 and range 31.44–39.01 kg/m^2^) and 7 male (mean BMI = 32.9, SD = 2.35 and range 31.19–37.4 kg/m^2^) participants with obesity. Gender was specified by the participants themselves in the online questionnaires prior to the testing.

Subjects were asked not to eat anything, not to smoke tobacco, and to drink only water one hour before testing to minimize potential influence on taste perception. At the beginning of the test, the participants were informed verbally and in writing about the study procedure and had to sign an informed consent form on site. All participants were monetarily compensated after the completion of the study and had the right to withdraw from the study at any moment. The study was approved by the ethics committee of the University Hospital Erlangen (project number: 128_21 B) and complied with the revised declaration of Helsinki. All participants signed a written informed consent form and data security statement prior to inclusion into the study.

### Stimuli and apparatus

To achieve taste stimulation, we used four aqueous solutions archetypical for basic tastes such as bitter, salty, sweet and umami. In addition to basic tastes, a more complex mango flavor stimulus that was of different viscosity and contained a fruity aroma was added to the stimulus pool. The complexity of the mango-flavored stimulus, included in the study, offers the opportunity to explore potential differences in how cognitive load affects pure taste stimuli versus flavor stimuli, and facilitates comparisons with other studies employing multisensory food stimuli. Artificial saliva, a tasteless, commonly used in research solution, was added as a control stimulus and was used to rinse the palate after the tastants^21^ (see Table 2). Sour taste was not included due to technical limitations of the delivery apparatus and due to its low association with overconsumption resulting in obesity. Bitter taste was added to cover the whole spectrum of pleasantness since it is a modality that is commonly disliked. All solutions were freshly prepared using drinking water as a base every 72 h, stored at 4 °C between the experiments and taken out of the fridge approximately 1 h before the experimental session.

**Table 2:** Overview of the taste stimuli utilized in the study. Provided concentrations are for 1L.

Taste	Substance	Concentration unit	Origin
Bitter	Quinidine sulfate salt	0.24 g/L	Carl Roth GmbH, Karlsruhe, Germany
Salty	Sodium chloride	15 g/L	Rewe GmbH, Köln, Germany
Sweet	Sucrose	150 g/L	Rewe GmbH, Köln, Germany
Umami	Monosodium glutamate	36 g/L	Ajinomoto foods EU SAS, Paris, France
Mango flavor	Mango syrup	250 g/L	Giffard Ltd, Nantes, France
“Artificial saliva”	Sodium bicarbonate	0.92 g/L	Carl Roth GmbH, Karlsruhe, Germany
	Potassium chloride	0.105 g/L	Carl Roth GmbH, Karlsruhe, Germany

**Figure 1:**
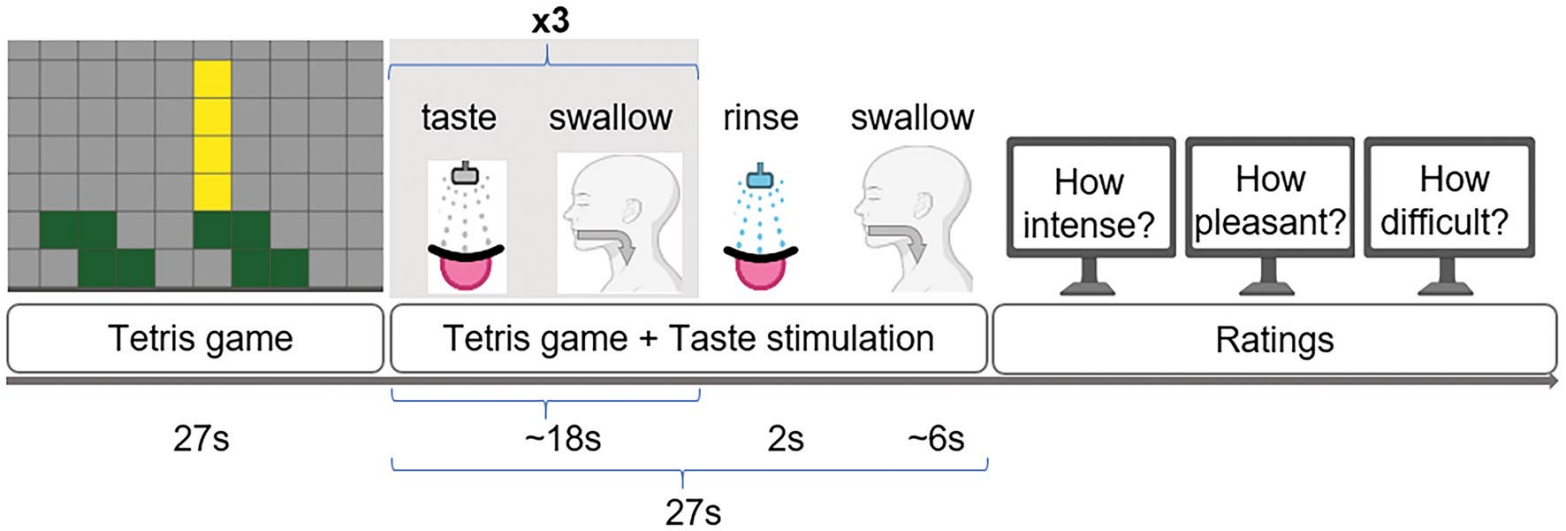
Schematic representation of a single trial. For the first half of a trial participants played Tetris following taste stimulation in the second half. One taste was presented 3 times followed by rinse and behavioral ratings.

To control the unintentional impact of uneven stimulus presentation, we ensured all gustatory stimuli were delivered to the participants in the same manner and at the same volume, by a modular computer-controlled low-pressure pump-system gustometer (Base 120, Cetoni GmbH, Korbussen, Germany)^22^. Solutions in 1 l bottles were connected to the 50 ml low-pressure syringes via inlet tubes. The outlet tubes were connected to the mouthpiece that ended in a straw-like tube that participants were asked to hold in their mouth during the experiment (Fig. 1).

### Procedure

#### Cognitive distraction task

The study employed the Tetris-game paradigm, as detailed by Schadll et al.^13^, to serve as the cognitive distraction framework. This framework introduced two levels of cognitive load, categorized as low and high, which were created by varying the speed of falling blocks in the game. Given that the speed of falling blocks was over twice as fast in the high cognitive load condition compared to the low cognitive load condition, this would lead to a higher visuospatial load, necessitating quicker decision-making and planning and execution of motor response in trials assigned to the high cognitive load condition.

### Participants instruction

Participants were initially not informed about cognitive load manipulation and were not told of the real purpose of the study to avoid manipulation of attention that could bias their responses. Instead, participants were asked to provide ratings of the taste stimuli they receive and try to play the Tetris game to the best of their ability. A debriefing session was held at the end of the experiment to inform participants about the study’s actual purpose.

### Experimental session structure

The experimental session lasted around 75 min and consisted of two parts: practice routine followed by main experiment.

### Practice routine

Practice part started with an assessement of subject´s ability to clearly perceive the taste stimuli used in the study. It is known that there is a substantial interindividual differences when it comes to taste sensitivity, especially of a bitter taste^23^. Thus, by mean of q-tips soaked in the taste solutions used for the study, we conducted an identification task of tastants including water. Initially, participants were asked whether they perceived any taste. If they indicated they could taste something, they were then prompted to choose the specific taste from a provided list of options. Participants who could not identify any taste were excluded from the subsequent phases of the study.

After the sensory assessement, a short practice run, which goal was to familiarize participants with navigation in the Tetris game and introduce taste application via gustometer took place. During this routine, participants were first presented with one taste stimulus for 2 s. Each stimulus was applied twice in the pseudorandomized order to avoid direct repetitions of the same taste. After each delivery of the stimulus, participants were asked to provide ratings for taste intensity and pleasantness. After the ratings, automatic rinse with artificial saliva was used for 2 s to rinse the palate. The stimuli, the setup of application and the flow rate were exactly as in the main part. After taste stimulation, participants were given instructions of the Tetris game and played without a gustatory stimulation for 30 s. The data of the practice part was used to retrospectively identify and potentially exclude participants that would correctly “guess” correct responses for the taste identification test, but whose intensity ratings repeatedly indicated no sensation of the study stimuli. No participants were excluded based on this criterion.

### Main experiment

In the following main part of the study, participants were instructed to play the Tetris game while they were presented with gustatory stimuli. Ratings of taste intensity and pleasantness as well as perceived difficulty of the Tetris game were obtained after each trial. We used a 100-point visual analogue scale for each of the ratings (intensity: 0 = no sensation, 100 = extremely high; pleasantness: 0 = highly unpleasant, 100 = highly pleasant; difficulty: 0 = extremely easy, 100 = extremely difficult). During each trial, participants first played the Tetris game without gustatory stimulation for 27 s and then another 27 s with stimuli applied in the manner described earlier. Only one taste modality (e.g., sweet) was presented during one trial. Such experimental design assured the immersion effect of the Tetris game prior to the gustatory stimulation and helped to increase interstimulus interval to avoid habituation of the gustatory system. There were 48 trials that lasted approximately 60 min in total. After every 12 trials there was a small optional break and participants could proceed with the game by a button press. There were 4 trials of low and 4 trials of high cognitive load setting for each taste stimulus. The order of the stimuli applications was pseudorandomized to assure no direct repetition of the same taste modality and equal number of applications. During each trial taste stimulus was delivered three times for 1 s with 1.5 ml/s flow rate intercepted by the swallowing time of approximatelly 5 s. After the third taste application, the rinse of the palate by artificial saliva was applied for 2 s with 1.5 ml/s. Subjects were provided with drinking water and were allowed to drink after the trial ended.

### Experimental details

Since there is evidence suggesting that satiety influences taste perception^24,25^, we collected ratings on hunger level in the beginning of the practice part and after the very last trial of the main experiment. For that we implemented another 100 mm visual analogue scale with 0 = not at all hungry and 100 = very hungry.

Trials with artificial saliva were used as a control condition. For the whole duration of the experiment, participants wore headphones playing white noise to mask the noise of stimuli delivery since the gustometer was placed in the same room. The experiment was designed and executed using PsychoPy3 software^26^. A keyboard was used to navigate the Tetris game.

### Physiological measurements

Together with behavioral performance, the physiological parameters such as skin conductance response (SCR) were recorded during the study. Multiple studies have reported that cognitive load triggers physiological changes that are discernible through the analysis of the skin conductance response^13,27^. We thus employed SCR measurement alongside the behavioral ratings to confirm the efficacy of our cognitive load manipulation, expecting to observe elevated skin conductance response values during high cognitive load compared to low cognitive load conditions. To record SCR two electrodes (LT118F GSR Finger Electrodes, ADInstruments Ltd., Oxford, UK) were placed on the palmar surface on the middle phalanx of index and middle finger of the left hand. Data was recorded via LabChart Pro v8.1.19 (ADInstruments Ltd., Oxford, UK) software and by PowerLab model 4/26 with FE116 GSR Amp (ADInstruments Ltd., Oxford, UK).

### Behavioral data analysis

In addition to collecting ratings on taste perception from the participants, we also tracked their performance in the game of Tetris. This performance was quantified by counting the number of rows successfully cleared in each Tetris trial, which we referred to as the “Tetris Score”. The aim of this assessment was to use the Tetris game’s difficulty ratings and the Tetris scores obtained under two different cognitive load conditions as variables to validate our cognitive distraction paradigm. To evaluate how these two variables behave under different cognitive load conditions, we computed a linear mixed model with fixed factor cognitive load (low, high) and the random factor of participants for difficulty ratings and Tetris scores separately.

To test the effects of cognitive load on sensory perception of gustatory stimuli, we computed two separate linear mixed models on intensity and pleasantness ratings with fixed factors cognitive load (low, high), taste (flavor, bitter, sweet, salty or umami), group (lean or overweight/obese) and gender (female or male) as well as the random factor of participants. Since our overweight/obese group consisted of fewer females in comparison to lean group, we added gender as factor to reveal its potential impact on intensity and pleasantness ratings. To control potential individual differences that might lead to unequal intensity perception across stimuli, and to reveal potential group and gender differences we included taste as a factor as well. Multiple comparison correction was applied to the *p*-values obtained from the mixed linear model analyses using the Bonferroni method and corrected estimates are reported throughout the manuscript.

Note that artificial saliva taste was excluded from the analysis after being tested separately as control condition. We used a dependent t-test with the intensity and pleasantness ratings of the artificial saliva condition for low and high cognitive load as outcomes (as our control condition). A dependent t-test was conducted on satiety ratings obtained in the beginning and at the end of the experimental session. To reveal potential intensity alterations related to the habituation and adaption of gustatory stimuli, we compared intensity ratings of the first 28 trials to the last 28 trials performing two-way ANOVA with fixed factors of order (first, second half) and taste.

To investigate a potential link between provided intensity and pleasantness ratings and the BMI, we ran a correlation analysis using Pearson´s coefficient.

The ⍺ level was set to 0.05. Note that all graphs depict one standard deviation.

### Physiological data analysis

During the experiment, precise time of each event like start of Tetris round, each stimulus application and trial end was saved by the PsychoPy software. These timestamps were then used to extract the continuous electrodermal activity (EDA) signal from the LabChart software in event-related fashion. Data from 9 participants was of low quality and not suitable for further analysis due to technical issues during recording. As window of interest, we selected 26 s before and after the very first stimulus presentation in a trial, which covered time with and without gustatory stimulation. A Python-based program was written to allow reading and manipulation of physiological data. We further used an open-source Python package NeuroKit2^28^ to decompose SCR to phasic and tonic components. Obtained values of tonic skin conductance level were then submitted to the linear mixed model with factors cognitive load (low or high) and taste (flavor, bitter, salty, sweet or umami) with the random factor of participants. The threshold for this test was set for *p* < 0.05 as well.

## Results

### Distraction-induced effects

**Figure 2:**
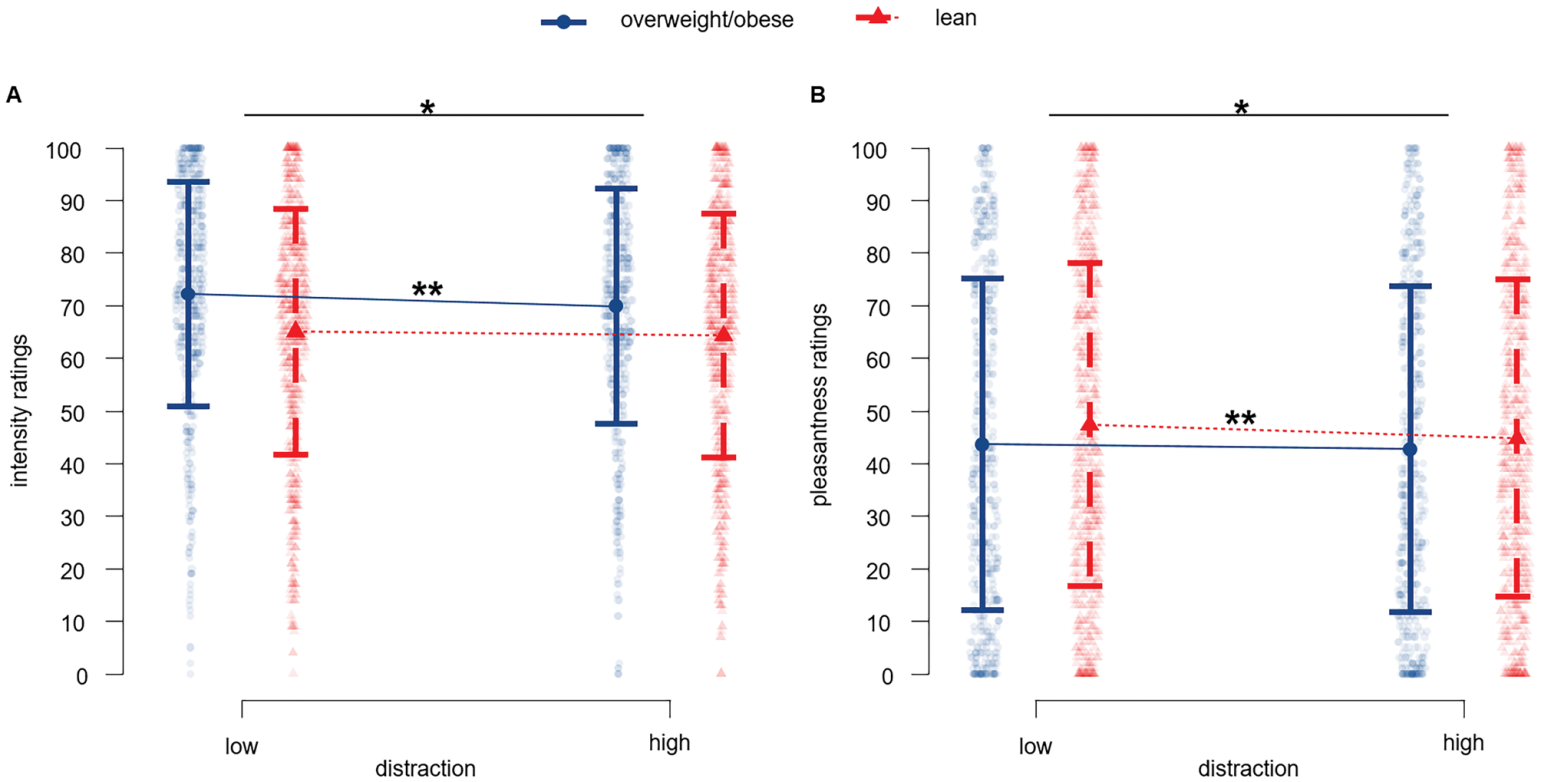
Effects of distraction on gustatory perception. (**A**) Intensity and (**B**) pleasantess decreases with rise of distraction level. Individual rating responses marked as faded dots, vertical line represents standard deviation, colouring scheme represents participants group of weight status. *Indicates *p* < 0.05, **indicates *p* < 0.01.

The linear mixed model on intensity ratings showed a simple main effect of *cognitive load* [F (1, 104.16) = 4.98, *p* = 0.017], *group* [F (1, 54.03) = 4.02, *p* = 0.050] , *gender* [F (1, 54.03) = 9.29, *p* = 0.004] and *taste* [F (4, 57.56) = 40.294, *p* < 0.001]. No significant interaction effects were revealed. As expected, there is a decrease in intensity perception under high (M = 68.4, SD = 22.9) in comparison to low cognitive load (M = 69.9, SD = 22.6) across the entire study population. The decrease in intensity perception under high cognitive load is more evident in overweight/obese group (low-to-high load: M = 72.2–69.8, SD = 21–22.1, *p* = 0.01) in comparison to changes observed in the lean group (low-to-high: M = 65.3–64.1, SD = 23.3–23.1, *p* = 0.3). Moreover, males exhibited stronger decrease of intensity perception (low-to-high: M = 64.09–61.29, SD = 23.22–22.8, *p* = 0.025) compared to female participants (low-to-high load M = 73.45–72.57, SD = 20.96–21.96, *p* = 0.348) (Fig. 2).

On average, stimuli were rated intermediately intense (all M = 67.7), yet not all stimuli were perceived as isointense. In line with “flavor enhancement effect”—the perceived intensity of the flavor is often greater than the sum of the individual taste components—mango drink (flavor stimulus) was perceived as the most intense (M = 78.76, SD = 16.69, *p* < 0.001), umami in opposite was rated as least intense and had the second biggest interindividual variability (M = 58.34, SD = 25.31, *p* < 0.001). Bitter and sweet were rated similarly intense (M = 64.86, SD = 25.61 and M = 66.22, SD = 20.47), but significantly less intense compared to salty (M = 70.30, SD = 19.68, *p* < 0.001 and *p* = 0.033 accordingly).

Across both cognitive load conditions and across all stimuli, the participants in the overweight/obese group perceived the intensity significantly higher (M = 71.02, SD = 21.83) in comparison to lean participants (M = 64.64, SD = 23.25; mean difference = 6.38, t = 6.777, *p* < 0.001). Moreover, female participants perceived all stimuli as more intense (M = 73.01, SD = 21.44) comparing to male participants (M = 63.02, SD = 22.95; mean difference = 9.9, t = 10.753, *p* < 0.001).

**Figure 3:**
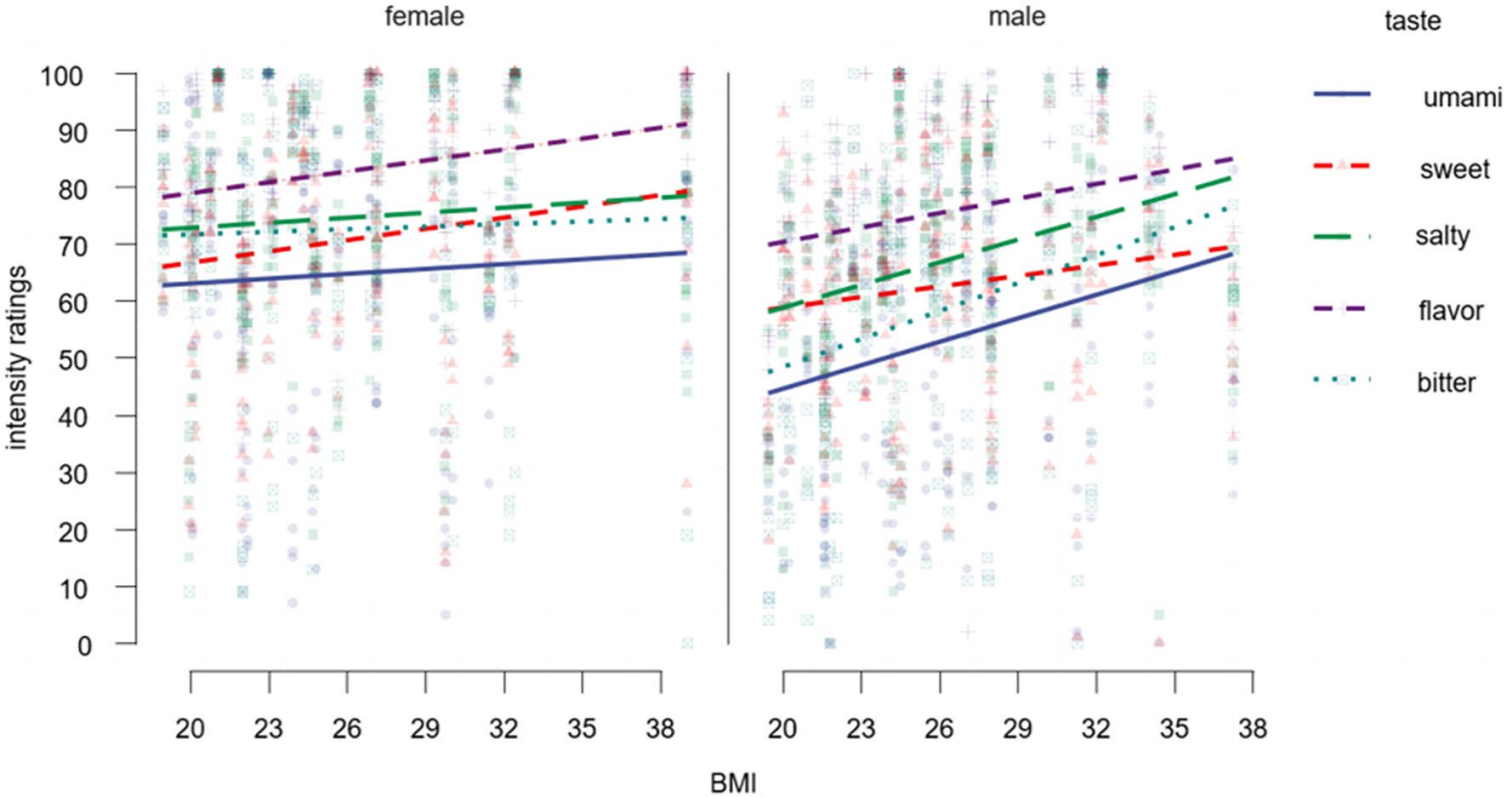
Correlation plot depicts changes in intensity perception as function of body mass index. Positive correlation depicted across genders. Colored scatter plots represent individual ratings across stimuli and horizontal lines depict mean values.

To access the potential link between the participants´ BMI and provided ratings for intensity, we performed Pearson´s correlation analysis between these two factors. The results show significant positive corerlation (r = 0.160, *p* < 0.001) pointing that with the rise of BMI the taste sensitivity increases. Moreover, the effect was modulated by gender, the stronger positive correlation of BMI and intensity perception was observed for every taste modality among male participants (all tastes r = 0.227, all tastes *p* < 0.001; all tastes female r = 0.101, all tastes *p* < 0.001). In female participants post-hoc comparisons across taste modalities revealed significant positive correlation between BMI and intensity ratings only for sweet (r = 0.175, *p* < 0.001) and mango flavor (r = 0.232, *p* < 0.001) stimuli (see Supplementary Table 1) (Fig. 3).

**Figure 4:**
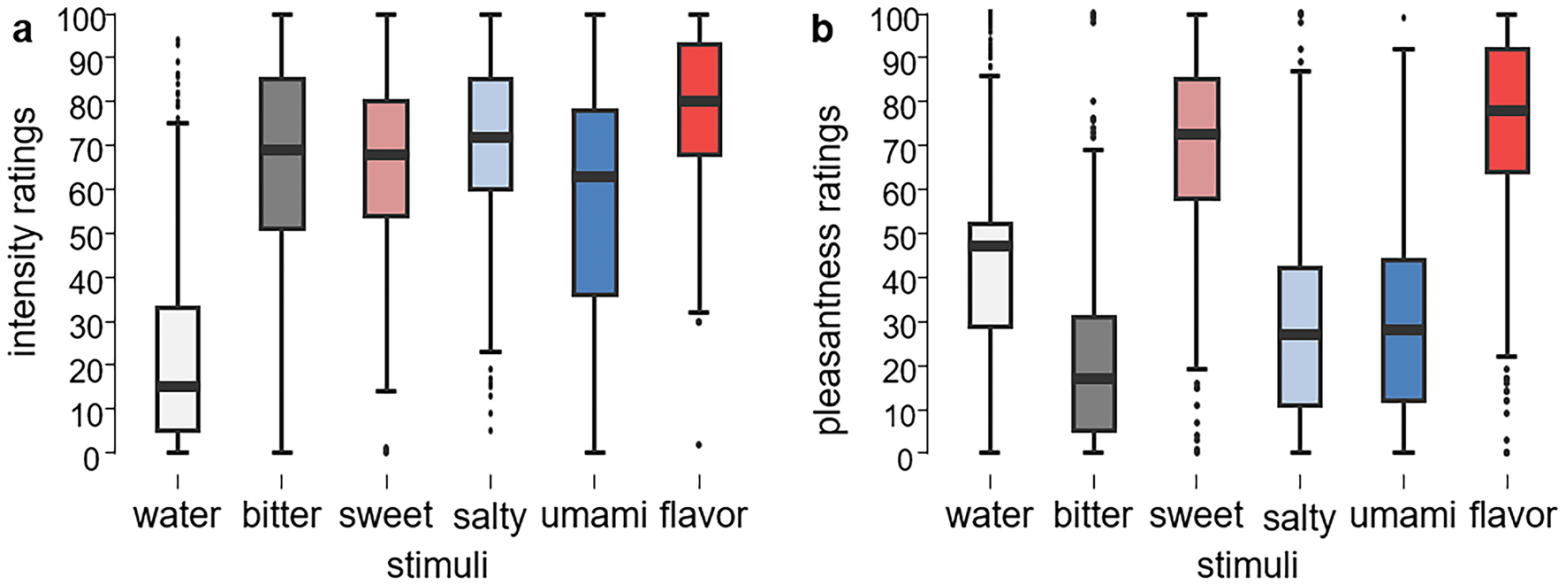
Box plots showing perception of gustatory stimuli averaged across both cognitive load conditions: (**a**) intensity and (**b**) pleasantness ratings. Water label on x-axes represents control condition with artificial saliva. Similar to intensity ratings, we observed high interindividual variability in preferences (e.g., bitter was rated as highly pleasant and sweet as aversive by some participants).

When investigating changes in pleasantness ratings, results revealed main effect of *cognitive load* [F (1, 67.15) = 5.77, *p* = 0.022] and *taste* [F (4, 54.09) = 40.29, *p* < 0.001] as well as interaction effect of *taste x group* [F (4, 54.09) = 4.44, *p* = 0.003] and *taste x gender* [F (4, 54.09) = 3.08, *p* = 0.023]. Pleasantness ratings decreased under high (M = 43.76, SD = 30.5) in comparison to low cognitive load (M = 45.63, SD = 31.11). While we observed a decline in pleasantness perception with the increase of cognitive load in both lean (low-to-high load changes: M = 47.33–44.76, SD = 23.18–23.33, *p* = 0.01) and overweight/obese group (low-to-high load changes: M = 43.83–42.38, SD = 21.09–22.11, *p* = 0.4), only the changes in the lean group differed significantly. As expected, not all stimuli were rated similarly in terms of pleasantness. As hypothesized, mango flavor was rated as the most pleasant stimulus (M = 74.47, SD = 21.61, *p* ≤ 0.001 to each other taste) followed by sweet taste (M = 69.09, SD = 21.08, *p* < 0.001 to bitter, umami, salty). Bitter was perceived on average as aversive (M = 20.26, SD = 17.29, *p* < 0.001). Salty and umami were rated similarly (M = 29.58, SD = 22.37 and M = 29.86, SD = 22.42) and were rather unpleasant to most participants (Fig. 4).

**Figure 5:**
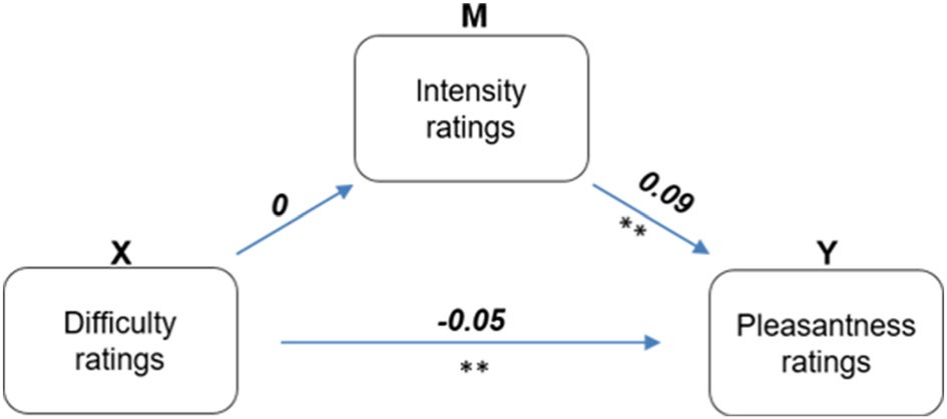
Mediation analysis model plot. X, predictor; M, mediator; Y, outcome. Values on arrows are non-standardized regression estimates. **Indicates *p* < 0.01.

The interaction *taste x group* effect was driven by significant differences in sweet perception between the groups. Lean participants perceived sweet taste as more pleasant (mean difference = 12.5, t = 6.14, *p* < 0.001, Tukey). Resolving the interaction effect between taste and gender revealed that female participants perceived flavor as more pleasant (mean difference = 6.79, t = − 28.194, *p* < 0.001) and salty (mean difference = − 9.91, t = − 5.47, *p* < 0.001) and umami (mean difference = − 14.32, t = − 7.43, *p* < 0.001) as less pleasant. We further investigated a link between BMI and pleasantness rating by computing correlations across taste modalities and gender (see Supplementary Fig. 1). Results revealed that sweet taste pleasantness was negatively correlated with BMI across both genders (male: r = − 0.184 , *p* = 0.004; female: r = − 0.326 , *p* < 0.001). In contrast, pleasantness ratings of salty taste positively correlated with BMI in male participants (r = 0.167, *p* = 0.010), while pointing in opposite direction across female participants (r = − 0.143, *p* = 0.036). No further statistically significant correlations between BMI and pleasantness ratings were present. Given that alterations in subjective intensity perception have the potential to influence perceived pleasantness, our subsequent analysis aimed to ascertain whether changes in pleasantness were genuinely prompted by cognitive distraction rather than merely being a result of altered intensity perception. Through regression-based mediation analysis in the context of maximum likelihood estimation, we examined both the direct effects of difficulty ratings on pleasantness and the indirect effects (mediation via intensity ratings). Utilizing a bias-corrected percentile bootstrap method with 10,000 replications^29^, we estimated the effects and their 95% confidence intervals (CI). We observed the significant total effect of difficulty ratings on pleasantness ratings (estimate = − 0.052, SE = 0.02, 95% [− 0.097, − 0.008], *p* = 0.015). The mediation analysis revealed a non-significant indirect effect of intensity on pleasantness ratings (estimate = 0, SE = 0.001, 95% CI [− 0.003, 0.003], *p* = 0.970), while significant direct effects of difficulty ratings were observed (estimate = − 0.052, SE = 0.02, 95% [− 0.097, − 0.007], *p* = 0.015) (Fig. 5).

### Validation of cognitive load paradigm and control variables

To validate that the utilized paradigm indeed elicited two distinct levels of cognitive load, we ran a linear mixed model on subjectively perceived difficulty of Tetris trials and on objective performance reflected in the number of successfully solved rows during Tetris game.

**Figure 6:**
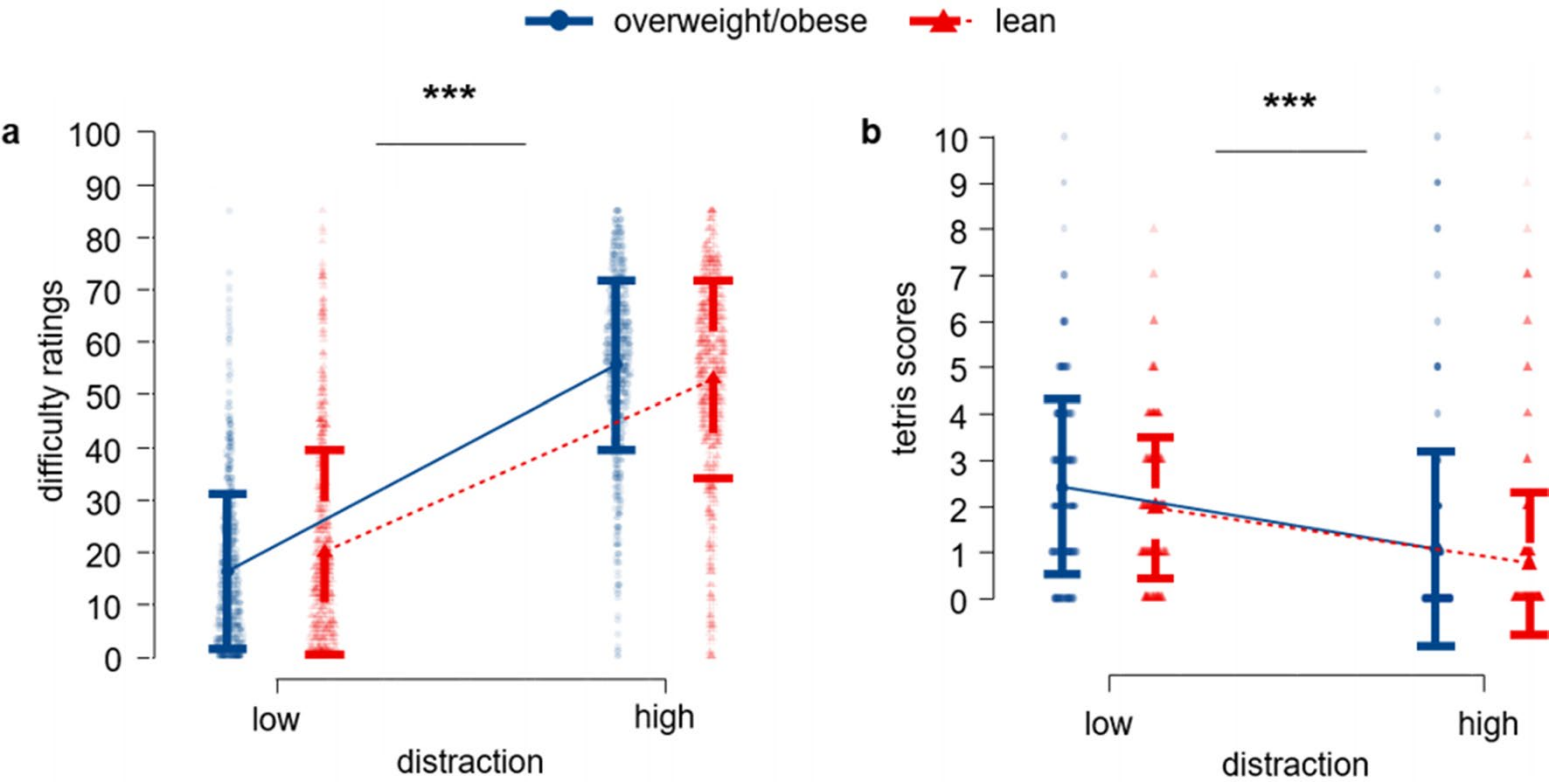
Perception of the high and low cognitive load condition of the Tetris game: (**a**) number of rows solved during Tetris game, (**b**) difficulty rating by the participants **indicates *p* < 0.01, ***indicates *p* < 0.001.

Results suggest the participants indeed perceived Tetris trials assigned to high cognitive load as more difficult [F (1, 55.86) = 248.59, *p* < 0.001, mean difference = 42.44] and respectively their performance dropped significantly under high cognitive load ([F (1, 54.55) = 110.819, *p* < 0.001, mean difference = − 1.26] (see Fig. 6).

As revealed by the linear mixed model on global mean values of the skin conductance level, participants had a statistically significant higher level of electrodermal activity during high load trials in comparison to low load trials [F (1,48) = 15.42, *p* < 0.001]. Skin conductance level increased from 2.59 on average (SD = 5.68) for low cognitive load to 3.17 on average (SD = 6.14) for high cognitive load.

We then tested whether intensity and pleasantness of the control condition was affected by cognitive load (high, low). One-way ANOVA revealed no statistically significant changes either in intensity ratings (F (1, 458) = 1.72, *p* = 0.18) nor pleasantness ratings (F (1, 458) = 0.03, *p* = 0.85) when comparing responses provided for artificial saliva taste under high and low cognitive load across both groups.

Satiety was rated differently in the beginning (M = 31.43, SD = 28.03) and at the end of the study (M = 55.17, SD = 20.13) [t (1,57) = − 5.24, *p* < 0.001]. Yet, it did not result in significant differences in intensity ratings of gustatory stimuli in the first (M = 68.22, SD = 22.47) and second half (M = 67.18; SD = 23.12) of the experimental session [F (1, 2763) = 1.209, *p* = 0.27]. Ratings of pleasantness were also not altered with the progress of the experiment (t (1, 458) = 0.24, *p* = 0.61) as revealed by values from the first (M = 44.27, SD = 21.97) and second half (M = 43.23, SD = 22.99).

## Discussion

This study delved into the intricate relationship between cognitive distraction and gustatory perception, shedding light on how intensity and hedonic evaluation of taste stimuli is affected when individuals are engaged in cognitive tasks. To utilize distinct levels of distraction (low and high), we manipulated the difficulty of the Tetris game paradigm and participants were asked to play while different taste stimuli were automatically delivered to their mouth. Our results demonstrate that cognitive distraction indeed affects the way taste stimuli are perceived that has potential to influence food choices and shape eating behavior^4^.

The findings of this investigation align with results of prior research (e.g.^9,11,30^) and our initial hypothesis, demonstrating that as cognitive load increases, the perceived intensity of gustatory stimuli diminishes. This phenomenon can be attributed to the limited capacity of attentional resources, which become occupied by the cognitive task at hand, hindering sensory information processing and preventing a proper monitoring of satiety clues that in turn leads to overconsumption. Yet, research exploring the relationship between taste intensity and subsequent food consumption does not consistently reveal a clear link between the two^31,32^.

Importantly, a notable aspect of this study is its focus on people affected by overweight and obesity, a group with a high prevalence worldwide. This expands prior findings by revealing a more pronounced decrease in intensity perception under high cognitive load in this group compared to lean participants. Several factors may contribute to these effects, including altered processing of gustatory stimuli and neural responses to the gustatory cues in areas responsible for decision-making, memory processing and stimuli inhibition, as well as lower working memory performance commonly observed in individuals with obesity^17,33,34^. Moreover, the use of a Tetris game, which also engages working memory, may have compounded the decrease in gustatory perception, particularly in the overweight/obese group. Furthermore, our study design required participants to provide the intensity ratings not immediately after each stimulus presentation, thus they had to actively maintain the sensory information that could have led to an even greater decrease of gustatory perception in overweight/obese group. However, not only taste sensitivity but the hedonic values of taste play a pivotal role in influencing food preferences and selection as well as overall eating pattern and is modified by obesity^35,36^.

For the first time, this study extends the understanding of distraction’s impact by showing that pleasantness perception of gustatory stimuli also changes with increasing cognitive load. Remarkably, even aversive taste stimuli, such as bitter and unpleasantly perceived salty taste, were rated as less pleasant under high cognitive load, despite being perceived as less intense at the same time. This aligns with previous findings on the dissociation between taste intensity and pleasantness and, supported by results of the mediation analysis, emphasizes that cognitive distraction effects on pleasantness are independent of intensity. A well-established phenomenon is an ability of diverse taste modalities to elicit similar pleasantness responses which implies that hedonic reactions are more closely tied to overall palatability than the specific sensory attributes of each tastants. It thus seems probable that distraction modulates hedonic reactions as a whole, implying that pleasantness should be investigated as an independent crossmodal construct within the context of distracted chemosensory perception.

However, the implications of altered pleasantness on actual food consumption remain uncertain, given a complex interplay of psychological, physiological and contextual factors shaping eating patterns. One potential scenario is that decreased palatability of food items could prompt seeking more palatable or rewarding food as a compensation mechanism resulting in overeating^37^.

Notably, when analyzing groups independently, a greater decrease in pleasantness ratings was observed in the lean group, while the same trend was not statistically significant in the overweight/obese group. Importanly, ignoring distraction effects, all taste stimuli but sweet were similarly rated between experimental groups. A speculative assumption is that diminished pleasantness might be a form of inhibition control in a distraction-rich food environment, preventing overeating by reducing overall palatability.

Despite inconsistencies, obesity has often been shown to associate with a poorer executive function and weaker inhibitory control along with a heightened reactivity of reward response towards food items and greater prevalence of food consumption beyond metabolic needs, hedonic eating^33,38,39^. Thus, it is probable that pleasantness of taste stimuli may dominate attention and generate higher salience rendering it more resilient to the top-down modulation of distraction compared to the lean group. This dual mechanism may explain differences in distraction-related effects between the experimental groups, highlighting the complexity of neurocognitive mechanisms of distracted eating. However, there is limited understanding, particularly regarding potential biomarkers for top-down distraction modulation and the neural mechanisms orchestrating observed behavioral changes. On the neural level, Duif et al.^9^ showed distraction to attenuate functional connectivity between primary and secondary taste cortices, that among others are responsible for processing pleasantness of taste stimuli. However, the authors did not obtain pleasantness ratings during the testing hence discarding the opportunity to capture the potential changes in hedonic values as function of distraction and preventing an assocication of behavioral with neural findings.

Despite observed changes in intensity and pleasantness being statistically significant, the magnitude is rather small partially due to high interindividual differences common for chemosensory perception. We also observed that some participants seem to be more susceptible to distraction-induced effects, but they do not appear to form a cohort based on one common criterion. This is in line with prior reports and highlights importance of future studies focusing on the features making individuals more susceptible to distraction-attenuated food perception resulting in altered consumption^5,9^. One possible explanation could be where attention was directed to, some participants might have concentrated on the Tetris game more, while others could have placed sensory stimuli in the spotlight. Since participants were not explicitly instructed to direct their attention either to taste perception or to primary concentrate on the Tetris game that enabled to switch the focus freely as they would in a daily life situation when confronted with a cognitive task during food consumption.

Ignoring distraction modulated effects, participants with overweight/obesity demonstrated higher sensitivity to all stimuli, while perceiving only sweet taste as notably less pleasant. It is hard to align the findings with prior research since results were often pointing in different directions. For example, participants with excess weight have been reported to have higher taste sensitivity, especially for sucrose and salt^40,41^ but also shown decreased sensitivity to sweet and salty taste^42^ or no differences in taste sensitivity at all^17^.

Crucially, gender-driven differences in sensitivity to gustatory stimuli and distraction-related effects were established within this experiment. Without distraction, female participants exhibited higher gustatory sensitivity that did not result in the noticeable differences in pleasantness perception, while male participants were more sensitive to distraction and were associated with higher decreases in ratings of intensity and pleasantness. Altogether, this factor, previously omitted in the literature on chemosensory perception under distraction, underscores the need for a balanced sample selection in future studies.

It is noteworthy that many prior studies have utilized complex flavor stimuli, such as milkshakes or real food items like quiche and M&Ms. In these studies, it remained uncertain whether the taste aspect itself was affected by distraction, or if the observed effects were driven by changes in the sense of smell, which plays a crucial role in food perception. Some research has suggested that distraction-related effects might be attributed entirely to alterations in the olfactory modality, impacting both behavior and neural responses (as demonstrated by^12,13^). To address this uncertainty, our study was designed to disentangle and isolate the gustatory modality from other sensory influences. We achieved this by using odorless aqueous taste solutions as the primary stimuli. However, to enhance the comparability of our findings with those of other studies and real-world food items, we diversified our stimulus set by including a mango drink. This mango drink not only contributed to the taste component but also introduced a fruity aroma, color, and varying viscosity to the stimuli, providing a more holistic representation of a flavor. By means of computer-controlled gustometry we ensured a uniform manner of stimulus delivery to the participants that allowed to avoid potential variables influencing ratings of intensity and pleasantness (e.g. different volume of consumed stimuli, unequal time between the stimuli etc.). Yet this may limit the comparability of the study’s results to real-world scenarios. Since actual food intake under distraction is out of this study scope, it remains unclear if decrease in intensity perception indeed leads to increased consumption or preferences in favor of stronger flavors, effects reported by other studies^6,11,43^.

In this study, we implemented a cut-off BMI of 40 kg/m^2^ since it was challenging to find participants with higher BMI and without other medical condition that might affect the results and/or be restricting participants from taking part in this study. This decision and a not large enough sample size, however, does not allow us to conclude if the distraction-related changes linearly progress with rise of BMI, since we did not have enough participants to form well defined clusters for a suitable analysis. Additionally, one could argue that by selecting participants with obesity but lacking its common comorbidities our results might not be representative for the broader population with obesity. Furthermore, pooling individuals with obesity and overweight into the same group may have influenced the outcomes of this study, hindering the ability to discern how the effects of cognitive distraction evolve across participants with different BMI ranges.

This study unveiled only behavioral alternations induced by distraction on the gustatory perception and there is a clear need for imaging studies to shed light on the neural processing orchestrating the observed changes.

## Conclusion

In conclusion, this study provides valuable insights into the challenges that cognitive distraction poses to gustatory perception, emphasizing the importance of considering weight status and gender as potential factors moderating these effects. Importantly, study results underscore disparate responses to distraction in taste perception across weight categories. Specifically, increased cognitive load affects intensity perception of taste stimuli in participants with overweight/obesity, contrasting with altered pleasantness perception observed in normal-weight participants under cognitive load. Weight status and gender contribute to the variability among individuals in their susceptibility to distraction-induced changes in gustatory perception that are crucial to consider when designing interventions or dietary recommendations. Understanding why some individuals are more affected than others could help tailor strategies to promote mindful eating and healthier food choices to address the obesity epidemic and its associated health risks. Further research is warranted to explore the neural mechanisms underlying these changes and to determine their potential implications for actual food consumption preferences and behaviors. By examining both intensity and hedonic values of taste stimuli under distraction, the study takes a more comprehensive approach to understanding how cognitive load affects our perception of taste. This holistic perspective contributes to a richer understanding of the complex interplay between cognitive distraction and gustatory experiences.

### Supplementary Information

Supplementary Information.

## Data Availability

The dataset generated and analysed during the current study is publicly available in the Zenodo repository (https://zenodo.org/records/10207997). Physiological data collected in this study is secondary to the study focus and will be provided by the corresponding authors via email request.
