# Supplementary material for: The impact of cognitive distraction on gustatory perception in volunteers with obesity
Source: Sci Rep. 2024 Jun 20;14:14268. doi: 10.1038/s41598-024-64722-0 (PMC11190272; doi:10.1038/s41598-024-64722-0)
Supplement: Supplementary file 1 — Supplementary Information. [file 41598_2024_64722_MOESM1_ESM.docx]

| **Taste** | **Gender** | **Pearson´s r** | **P value** | |
| --- | --- | --- | --- | --- |
| bitter | male | 0.291 | <0.001 | *** |
| sweet | male | 0.139 | 0.029 | * |
| umami | male | 0.247 | <0.001 | *** |
| mango flavor | male | 0.217 | <0.001 | *** |
| salty | male | 0.299 | <0.001 | *** |
| bitter | female | 0.032 | 0.633 |  |
| sweet | female | 0.175 | <0.001 | *** |
| umami | female | 0.062 | 0.358 |  |
| mango flavor | female | 0.232 | <0.001 | *** |
| salty | female | 0.086 | 0.207 |  |

**The impact of cognitive distraction on gustatory perception in volunteers with obesity**

**Supplementary Table 1**| Statistical Analysis of Correlations Between Taste Quality Intensity Ratings and BMI Across Gender

* p < .05, *** p < .001

**
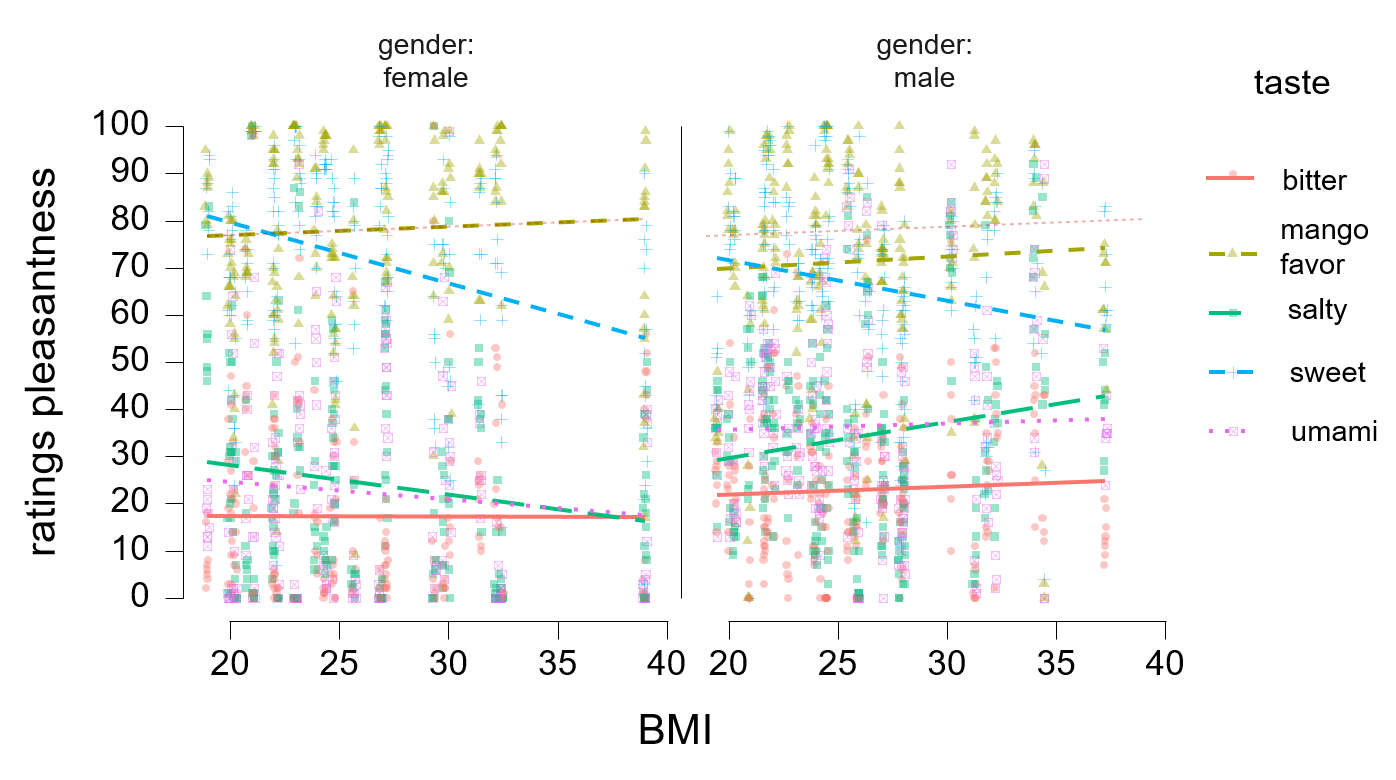
**

**Supplementary Fig. 1|** Correlation plot depicts changes in pleasantness perception as function of body mass index. Correlation depicted across genders. Colored scatter plots represent individual ratings across stimuli. Statistics for correlation coeficients see Sumplementary table 2.

**Supplementary Table 2**| Statistical Analysis of Correlations Between Taste Quality Pleasantness Ratings and BMI Across Gender

| **Taste** | **Gender** | **Pearson´s r** | **P value** | |
| --- | --- | --- | --- | --- |
| bitter | male | 0.0466 | 0.468 |  |
| sweet | male | -0.1840 | 0.004 | ** |
| umami | male | 0.0283 | 0.659 |  |
| mango flavor | male | 0.0491 | 0.444 |  |
| salty | male | 0.1650 | 0.010 | ****** |
| bitter | female | -0.0010 | 0.988 |  |
| sweet | female | -0.3262 | <0.001 | ******* |
| umami | female | -0.0910 | 0.183 |  |
| mango flavor | female | 0.0475 | 0.488 |  |
| salty | female | -0.1431 | 0.036 | ***** |

* p < .05, ** p <= .01, *** p < .001
